# Emotional Self-Care: Exploring the Influencing Factors Among Individuals With Cancer

**DOI:** 10.3389/fpsyg.2022.898345

**Published:** 2022-06-06

**Authors:** Ann Tresa Sebastian, Eslavath Rajkumar, Romate John, Monica Daniel, Allen Joshua George, Rajgopal Greeshma, Treasa James

**Affiliations:** ^1^Department of Psychology, Central University of Karnataka, Gulbarga, India; ^2^Humanities and Applied Sciences, Indian Institute of Management Ranchi, Ranchi, India; ^3^Department of Medicine, KMCT Medical College, Kozhikode, India

**Keywords:** cancer, emotional self-care behaviors, factors influencing emotional self-care, wellbeing, qualitative study

## Abstract

Cancer is a leading source of distress and fatality worldwide. Cancer-related aberrant cell proliferation causes excruciating pain and impairment. To cope with pain and manage symptoms and illness, pharmaceutical and non-pharmacological options are available. Self-care behaviors are recognized as a key source in symptom management and improving quality adherence to treatment among the current non-pharmacological strategies. The intervention measures to improve self-care were hardly impacted because of the narrow focus on physical self-care. Bringing in emotional self-care and addressing the individual's emotional health can enhance the effectiveness of interventions on a holistic level. Hence, this study has attempted to explore the factors that influence emotional self-care among individuals with cancer. Following an exploratory research design, the data were collected from 15 participants (4 men and 11 women) using purposive sampling and semi-structured interviews. Through thematic analysis, eight major themes were identified: physiological factors, social factors, family factors, psychological factors, individual factors, socioeconomic factors, cultural factors, and spiritual factors. The findings explained the emotional self-care among patients with cancer and how different identified factors influence their emotional self-care practices.

## Introduction

In today's world, non-communicable diseases (NCDs) and psychiatric illnesses are the most wellacknowledged health hazards. It impacts individuals and systems across the globe, especially making the developing nations vulnerable to these threats that are uncontrollable (World Health Organisation, 2018). NCDs are growing as the largest cause of death and disease across the globe (World Health Organisation, 2007), and the associated pain and disability that is inflicted on an individual, are also an increasing cause of poverty and distress in individuals (Allen and Feigl, 2017). Cancer is the fourth most prevalent non-communicable disease, with the highest rates of morbidity and mortality (World Health Organisation, 2014). In India, cancer is the second-leading cause of mortality in urban regions and the fourth-leading cause of death in rural areas (Smith and Mallath, 2019; Mathur et al., 2020).

Cancer can strike a person for a variety of reasons, including epidemiological shifts (He et al., 2020), psychosocial impacts (Shahab et al., 2018), and environmental and genetic factors (Al Ajmi et al., 2020; Oluwasanu and Olopade, 2020). The European Code Against Cancer (ECAC) has identified several cancer risk factors, including alcohol consumption, being an active or passive smoker, being overweight or obese, physical inactivity, poor dietary habits, increased ultraviolet radiation exposure, and human papillomavirus (HPV) infection (World Health Organisation, 2007; Shahab et al., 2018; Coates et al., 2020). Treatment modalities for cancer have evolved from extremely invasive to minimally invasive (Gotoda and Hatta, 2017). However, the after-effects and modifications made to the patients' bodies create agony and misery, resulting in their inability (Nipp et al., 2017; Granek et al., 2018; Carlson et al., 2019). As a result, the most effective method for reducing such discomfort and assisting patients in managing their symptoms is to engage in self-care.

Multiple physiological and psychological changes can be brought in individuals diagnosed with cancer when they follow a strict self-management technique (Whitaker et al., 2015). Self-care habits are thought to have more positive elements since they improve people's wellbeing, ability to move around, and help to keep healthcare coverage effective (Riegel et al., 2019). World Health Organization has devised several implementation tools to prevent non-communicable diseases, where they define “self-care as the ability that an individual or a group has, to promote health, prevent disease, maintain health, and cope with illness and disability with or without the support of a healthcare provider.” Prior shreds of evidence have found self-care itself as an important non-pharmacological aspect that helps to manage many chronic illnesses, including cancer (Rico et al., 2017).

Self-care for an individual can be done in many ways and dimensions, including physical self-care, emotional self-care, or even spiritual self-care practices (Goudarzian et al., 2017a). Physical self-care is the process in which the individual personally takes care of oneself and optimizes physical function and keeps safe from all unhealthy activities. Zimmermann et al. (2018) reported that physical self-care methods can effectively control somatic symptoms and pain management, as well as a variety of psychosocial problems experienced by patients with cancer. Emotional self-care instills in the individual a belief that they can manage their symptoms (Qian and Yuan, 2012). Emotional self-care can be explained as maintaining a positive and compassionate view of the self, negotiating external and internal demands, identifying, accepting, and expressing a range of emotions (Dorociak, 2015). Among individuals diagnosed with cancer, emotional self-care can be an effective mechanism that supports them to preserve their self-confidence in overcoming the disease (Mesurado et al., 2018). Kawasaki et al. (2011) and Nasri et al. (2020) identified that emotional self-care helps the individual to rationalize their emotions and thoughts related to their diagnosis as well as help them to be prepared for the treatment, its side effects, and also to manage all their negative emotionality. With the help of emotional self-care, the individual acquires a transition from the state of fear to a state where they strive to accept the new condition and make new strategies to overcome the situation (López et al., 2021). By answering the question of why an individual succumbs to the practice of emotional self-care behaviors, there can be multiple reasons which includes their physical conditions and treatment processes, psychological makeup and mental states, influence from societies and families, socioeconomic conditions, etc. (Goudarzian et al., 2017b). Adaptation to new situations and the ability to cope with side effects and treatment-related changes can all be achieved with good emotional self-care (Bressi and Vaden, 2017).

According to the research, a greater emphasis is placed on understanding the various elements that influence physical self-care. Self-care has different dimensions, and integration of all those dimensions gives proper management of the symptoms and helps to improve the conditions and swelling of the patients (Goudarzian et al., 2017b; Wallace et al., 2020). Nasri et al. (2020) pointed out that there is a lack of theoretical understanding of emotional self-care among patient, and due to this, various self-care and self-management intervention that was conducted did not show the expected results (Mikolasek et al., 2018; Stoerkel et al., 2018). Johnson et al. (2018) reported that there was a significant increase in the quantity of self-care patients engaged during the first phase of the trial, but that these behaviors did not remain the same following the intervention. Further, they reported that interventions were ineffective because people oppose the emotional part and place a high priority on physical self-care. It is critical to maintain adequate control over emotional health, as this influences the impact of an individual's health behavior (Haghshenas, 2017; Arslan, 2018). Hence, this focuses on the different factors that influence emotional self-care, considering the significance of improving the overall wellbeing of patients with cancer.

## Methods

### Participants and Recruitment

The explorative design was used to conduct the study. The individuals diagnosed with cancer and undergoing treatments such as surgery, chemotherapy, radiation therapy, or other major medical management techniques were recruited using purposive sampling. Out of the 46 participants approached, 15 participants (4 men and 11 women) willingly gave their consent to be the part of the study. The major reason reported to decline in the participation was their ill-health condition. The sociodemographic characteristics of the participants are included in Table 1. The study focused on participants who had access to quality treatment methods. Therefore, data were collected from patients who were taking treatment in Tata Memorial Hospital, Mumbai. This hospital has been considered as one of the top hospitals in India where patients from different states come for treatment. The interview schedule was developed with an extensive review of literature; finally, a set of 17 questions were formulated and were verified by three experts, such as psycho-oncologist, oncologist, and researcher working in the area of psycho-oncology and health psychology. According to the suggestions and feedback, necessary modifications have been made and face validity of the interview schedule has been established.

**Table 1:** Sociodemographic characteristics of the participants.

**Variable**	***n*** **(15)**	**%**
**Age**		
20–35	6	40%
36–40	3	20%
41–55	2	14%
56–60	3	20%
61–75	1	6%
**Gender**		
Male	4	27%
Female	11	73%
**Educational qualification**		
Schooling	2	14%
Under graduation	7	47%
Postgraduation	6	40%
**Residence**		
Urban	14	94%
Rural	1	6%

### Data Collection Procedure

Prior to the data collection, hospitals have been approached to obtain permission to collect the data. As this study was conducted during the pandemic period, many of the hospitals did not give consent to conduct the study. So, social media platform has been used as a source to get the details of the patients with cancer. Initially, researcher identified a specific health group through Facebook, which includes healthcare professionals, cancer survivors, and patients with cancer working and taking treatment in the Tata Memorial Hospital, Mumbai. The main purpose of this self-help group is to share their experiences and support and psycho-educate each other. Later, the researcher introduced herself to a doctor of that group who is known, then, the doctor added the researcher to that group, and the researcher introduced herself and explained the purpose of the study and requested for the voluntary participation. After getting the contact details of the participants from the group, participants were approached individually. As this study was conducted during the pandemic period, many patients were not comfortable to participate in the study through a face-to-face interview. Therefore, the telephonic method was chosen to conduct the interviews. Before conducting the interview, their convenient time to participate in the interview was confirmed. During the process of interview, a good rapport and trust were established between the interviewer and respondent, and the purpose of the study was explained. It was made sure that the interview process was sufficiently long for the subjects to express themselves in detail. The researcher used a semi-structured interview schedule, and the same interview schedule was used to ask the questions to all the participants and tried to receive the apt responses related to the study objective. They were asked to clarify whether there is an ambiguity in the question been asked. The conversation was recorded with prior permission from the participants. The questions that were asked to the participants are as follows: “*What do you think emotional self-care can be? Can you elaborate on it?” or “Can you explain your opinion about the factors that influence your self-care practices by several negative feelings like fear, anxiety, depression, and feeling of uncertainty?.”* Some participants were encouraged to speak with the help of several questions, such as “Can you clarify a little more” or “Will it be possible for you to explain it with examples.” Further, after the completion of each interview, the recordings were transcribed by the first author and it has been cross-verified by other authors. The necessary suggestions were received from experts and made an effort to enhance the skills of analysis, and all the responses were recorded. After the data were analyzed by the first author, it was reviewed by the other authors.

### Data Processing and Analysis

**Figure 1:**
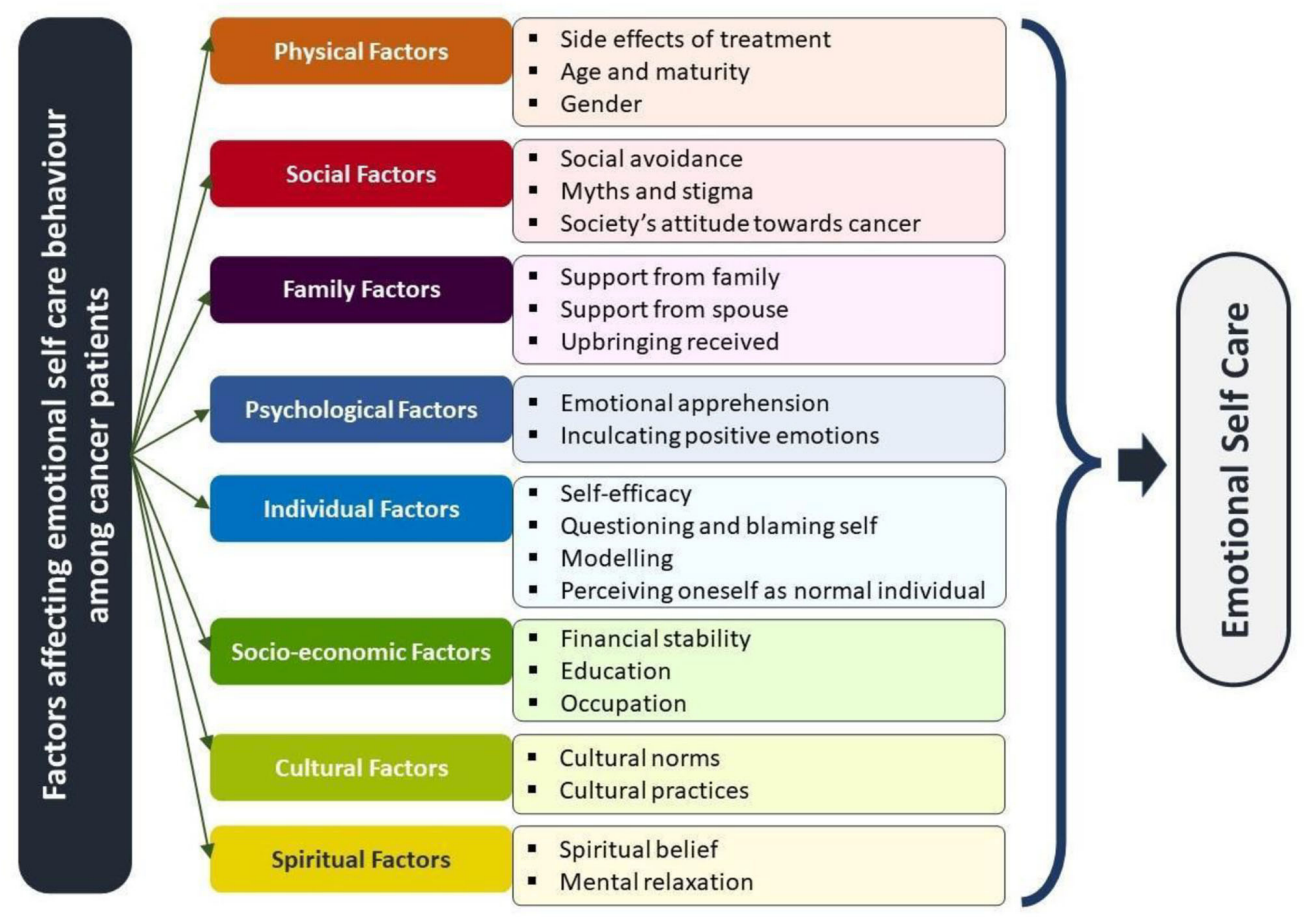
Explored factors that influence emotional self-care among cancer patients.

Interviews that were conducted were transcribed. In this study, Braun and Clarke (2006) concept of qualitative thematic analysis is used for data analysis. In the process of transcription, the recordings were listened to multiple times to ensure the familiarity of the information shared and underwent multiple amounts of scrutiny to ensure the information. Through the verbatims, primary-level codes were identified. During this process, the more frequently used codes which are close to the point of exploration of the objectives of this study were given more weightage. Further, different codes obtained from the data set were sorted into potential themes, different subthemes, and main themes. For each subtheme that was identified, categories and subcategories were very identified, to give much clarity to the findings. The identified theme and subtheme were reviewed to represent the data; later, the themes were named and defined, so they ideally summed up the essence of the data and addressed the research question directly. Based on these conclusions, the report was generated (Figure 1).

### Ethical Consideration

Informed consent was taken, and the study details were provided to participants before the interview. Participation was voluntary, and no remuneration was provided for the participants. The participants were briefed about confidentiality and their rights to withdraw from the study at any time during the study. Permission to record the telephonic interview and the confidentiality of the voice clip recorded were assured. At the end of the interview, debriefing of the information collected and the purpose of the study were mentioned to all the participants. The permission to conduct the study was obtained by the Department of Psychology, Central University of Karnataka.

## Results

From the information collected and analyzed through thematic analysis, eight major themes emerged (Tables 2, 3). These are (1) physical factors, (2) social factors, (3) family factors, (4) psychological factors, (5) individual factors, (6) socioeconomic factors, (7) cultural factors, and (8) spiritual factors.

**Table 2:** Themes, subthemes, and categories identified, their codes and *n*%.

**Themes**	**Subtheme**	***n*** **%**
Physical factor	Side effects of treatment	57%
	Age and Maturity	40%
	Gender	23%
Social factors	Social avoidance	30%
	Myths and stigma	17%
	Society's attitude toward cancer	53%
Family factor	Support from family	31%
	Support from spouse	23%
	Upbringing received	12%
Psychological factors	Emotional apprehension	47%
	Inculcating Positive emotions	50%
Individual factors	Self-efficacy	49%
	Questioning and blaming self	30%
	Modeling	28%
	Perceiving oneself as Normal individual	45%
Socio economic factors	Financial stability	46%
	Education	41%
	Occupation	37%
Cultural factor	Cultural norms	41%
	Cultural practices	22%
Spiritual factors	Spiritual belief	21%
	Mental Relaxation	16%

**Table 3:** Themes and subthemes identified.

**Objective**	**Theme**	**Subtheme**
**Factors influencing emotional self-care**	Physical factor	Side effects of treatment
		Age and maturity
		Gender
	Social factors	Social avoidance
		Myths and stigma
		Society's attitude toward cancer
	Family factor	Support from family
		Support from spouse
		Upbringing received
	Psychological factors	Emotional apprehension
		Inculcating positive emotions
	Individual factors	Self-efficacy
		Questioning and blaming self
		Modeling
		Perceiving oneself as normal individual
	Socio economic factors	Financial stability
		Education
		Occupation
	Cultural factor	Cultural norms
		Cultural practices
	Spiritual factors	Spiritual belief
		Mental relaxation

### Theme 1: Physical Factors

From the responses, one of the influential factors was the individual's physical abilities. Body and mind are connected entities; any disturbances in the physical makeup of the individual will show a change on an emotional level. The three subthemes that are identified include (1) side effects of treatment, (2) age and maturity, and (3) gender.

#### Side Effects of Treatment

When the body is exposed to high levels of radiation and other chemicals, there are several short- and long-term consequences. Some of the participants reported side effects, such as hair loss, skin patches and rashes, extreme mouth ulcers, lack of saliva, cessation of a body part, like the breast, and so on. This was found to have a negative impact on the practices of emotional self-care. For example,

…*…. went to get treated, he said (doctor) that you won't be getting saliva for your whole life. So on that day, I was very depressed; if I don't get saliva, the food pipe doesn't open, and if the food pipe does not open, I won't be able to eat food, then why am I living? …. (PARTICIPANT 5, AK)*

#### Age and Maturity

When it comes to emotional self-care, participants agreed that the individual's age and the maturity that comes with age play important roles. As they get older, their emotional experience grows, and they feel mature enough to care for themselves. Such life experiences, as they accumulate over time, will impact emotional self-care practices. For instance, the participants responded like,

…* So, if you are mature enough; you have to take care of yourself. If you are a child then you will have to see it…. (PARTICIPANT 2, RH)*

The “aging” process also determines their ability to tolerate pain and provides a perspective of maturity that influences an individual's ability to deal with a problem. For example, participant SIL reported that,

…* Age also influences because as you become more mature, even if you get a little bit of trouble, you will have the ability to bear it. (PARTICIPANT 7, SIL)*

The maturity that an individual gains through aging results in a positive change.

#### Gender

The gender with which a person identifies also demonstrates an influence on emotional self-care practices, as reported by the participants. Being recognized as a woman or man, which is a part of their self-identity, is seen to influence the patients regarding their outlook on pain and management of the conditions. For example, participant DS identifies,

*Women have a unique ability, they have a lot of feminine and motherly instinct in them that would eventually help them face much more… like something very usual, but the men find it quite difficult to face it.... (PARTICIPANT 10, DS)*

### Theme 2: Social Factors

Participants have stated that the attitude of society is very crucial to them. This theme was developed by analyzing participants' responses to the perceived presence and absence of social support received by the individual. Participants opined that society and family have an impact on their disease condition and self-care practices. The subthemes that are identified include (1) social avoidance, (2) myths and stigma, and (3) society's attitude toward cancer.

#### Social Avoidance

Participants reported that identifying and avoiding negative people and the environment helps them to stay away from the judgment of society, and irrelevant and incorrect information.

…*.People generally give examples of others in their journey of cancer and how all they deal with and fought over it. In my opinion, it's better to cut off and avoid such people. It's always better to keep relationships that you are confident about rather than having so many people who make you feel uncomfortable…. (PARTICIPANT 15, AM)*

Patients who avoided people who were influencing them in a negative way had a positive influence and were seen to engage in more positive emotional self-care behaviors.

#### Myths and Stigma

There are many myths and stigma associated with cancer due to the lack of awareness about the disease, which has a negative impact on emotional self-care behaviors. The majority of participants responded that this disease is considered as the person's Karma. The society views cancer as a natural death penalty for bad deeds, such as enjoying and living a life that the patient used to live. For example,

*People connect it with karma, ‘I did a lot of bad things in my previous life', and that's why I have it, they say ‘I used to spend and waste a lot of money going to pubs', and ‘this is happening to me because I used to enjoy my life'..…. (PARTICIPANT 10, DS)*

Some participants also explained that people consider that cancer is transmitted through touch; hence, they do not use the utensils used by them nor come near their house premises. In such cases, the families also experience a lot of stress and dilemmas. As reported by participant BK,

*People also face untouchability during the treatment. I know some people who have complained that no people are touching them because they have cancer..…*.

*(PARTICIPANT 6, BK)*

#### Society's Attitude Toward Cancer

Concerning myths and stigma, which are having a negative impact, participants also responded that when the people around them and the situation they are in foster support and hope, the patient feels external support and is motivated to engage in self-care behaviors. For example, participant RH reports,

*I used to stay in a building where we have a long term relationship with each other, when I meet them, that person will always tell me that I will pray for you to get well soon and all, so those kinds of words create hope, receiving support is essential when you undergo treatment..…. (PARTICIPANT 2, RH)*

However, when a patient is neglected by society and social support, it creates a hostile environment and emotions, causing them to exacerbate their condition. For example, participant SW reported,

*So, it is you who choose the circle and with whom you want to live. Near my house I have neighbors who are very negative. They won't come and visit me because they feel that if they come and see me, they will also become a patient...…. (PARTICIPANT 1, SW)*

Hence, the way society understands and approaches the patient also plays an integral role in the patient's life.

### Theme 3: Family Factor

Participants identified the importance of understanding the role of the family and the importance of each member, including the patient himself. As the family is a system that connects the individual to society, the responses and the support that the patient needs from the family to make a difference are also identified to be very important. A total of three subthemes are identified likewise to understand the family influences, which are, (1) support from family, (2) support from a spouse, and (3) upbringing received.

#### Support From Family

Participants responded that family support is an essential factor that helps the patient to focus on more of their issues. The strength that the family gives in terms of support and companionship helps the individual to face all the negative social influences. This support was seen to positively influence emotional self-care. As a source of support alongside the patient, the participants reported that they benefit individuals emotionally and feel the strength to fight cancer. For example, participant KM reports,

*My family is very important. So, when I can see so much love and support from my family, I feel that you need to go back and get better soon for them and that motivates me..…. (PARTICIPANT 9, KM)*

#### Support From Spouse

Participants reported that the support from their spouse also influences their emotional self-care practices. When such support is received from the spouse, it influences the patient more positively. Participants reported that if the partner can understand and support the patient throughout the treatment, it helps them with their emotional self-care practices.

*....my husband is like my friend, he is very deep, he knows that I'll win. He always encourages me to fight…. (PARTICIPANT 14, BJ)*

#### Upbringing Received

The way their parents raised the individuals, helps to deal with their situation and stress. Parenting impacts the emergence of emotional self-care practices, which they follow even before the diagnosis.

*For example, a similar view was shared by Participant AM and SIL*,

*.... the way I was brought up also influenced me. The personal things that we get from our home and our background also may influence us…. (PARTICIPANT 7, SIL)*

### Theme 4: Psychological Factors

This subtheme refers to the various psychological factors that influence the client's constructive participation in emotional self-care behaviors, and these factors can affect the patient both positively and negatively in such practices. There are majorly 2 categories in this subtheme, which are (1) emotional apprehension and (2) inculcating positive emotions.

#### Emotional Apprehension

Another factor that was identified to influence emotional self-care practices is the different emotional apprehensions that the patient faces. Their apprehension may be related to various feelings of fear, stress, and ambiguity about the future. A number of four categories were identified which include (1) fear of relapse, (2) negative emotions, (3) stress, and (4) uncertainty of the treatment.

#### Fear of Relapse

Participants reported that even when they are in remission, there is a chance that the disease will reappear in various forms or types. This fear instills anxiety in participants, affecting their emotional self-care practices and having a negative impact on their emotional self-care. For example, participant RH responded,

…*.. Many people fear the relapse of cancer. Again, this will lead to stress, anxiety, fearfulness, and again you will not be able to manage your emotions right…. (PARTICIPANT 2, RH)*

#### Uncertainty of Treatment

As participants highlighted, cancer treatment is both painful and expensive. Even after high intensities of radiation exposure, there is no assurance that the treatment will be completely effective for the patient. As a result, there is always some doubts in the participants' minds about whether their treatment will work or not.

*Even though we are getting the treatment, we are not sure whether our treatment will work or not, we are not emotionally healthy. So, if the treatment somehow doesn't work as it should, there will be an increasing impact on the emotional part because I myself experienced that…. (PARTICIPANT 3, NK)*

#### Negative Emotions

The patients experience a variety of negative emotions. This includes a range of worries and concerns about the health conditions, their future, their present psychological status, and physical support system. When they engage in such negative emotions and thoughts, their level of acceptance is called into question, and the intensity of these negative moods begins to rise, for example,

*One thing is definitely depression because it is like a chain reaction you think about one thing, it leads to another, and it goes on, so what one needs to do is, we need to snap out of that negative emotion once and look into the positive side even in your negative situations…. (PARTICIPANT 10, DS)*

#### Stress

As a patient, they will be subjected to various stressors. In this condition, most of the participants have reported encountering more distress than eustress; hence, this also gives a lot of negative emotional reactions to the occurrence of that. Majorly identified stressors were the physical changes they had to undergo due to the treatment and financial instabilities.

*Stressors are everywhere, and stress will influence your self-care when you allow it to influence you, if you deal with your stress you can handle it properly. Because stress comes when you don't handle your problems, and you make it a big issue, stress comes there…. (PARTICIPANT 1, SW)*

#### Inculcating Positive Emotions

Participants identified that being open to incorporating more positive emotions into their lives will aid them in their emotional self-care, allowing them to deal with their conditions in a constructive manner. In this subtheme, three categories are identified which are (1) emotional stability, (2) optimism, and (3) sense of gratitude.

#### Emotional Stability

Even though there are several negative and positive situations and conditions that the individual experiences, the ability to face all emotions and bring stability and confidence was identified as emotional strength. For example, participant DS responded

.... *If you are positive and emotionally stable, I think that would be enough. If you are emotionally in a good place, your emotional self-care also will be better. You become more positive about it…. (PARTICIPANT 10, SW)*

Being emotionally stable also aids in understanding and respecting one's own and other people's emotions. This has made a significant difference in the patient's emotional self-care practices and has positively impacted emotional self-care. Participants also reported that emotional stability is one of the keys helping them to manage their conditions on one's own

.... *So, if you are emotionally stable, you start understanding your emotions as well as others emotions, and also you'll understand why the other person is doing it…. (PARTICIPANT 12, KJ)*

#### Optimism

The participants identified that it is important that one must seek a positive attitude and have hope that they will be able to overcome the condition, and along with that, they also have to spread positivity to others as well. This helps them to positively influence others. Being so, the emotional self-care one takes also helps them to stand out, and through seeking a more positive outlook of the condition, their emotional self-care will be quite intact.

*.... I think positiveness is the most important thing. If I am suffering with the current procedure, I think I'll go for other procedures. It's okay. We have to. That is the most important thing in health care…. (PARTICIPANT 14, BJ)*

#### Sense of Gratitude

Participants reported that having a sense of gratitude for being able to receive treatment, adequate help and support is an important part of understanding and respecting. Even the smallest gesture of support that the patient receives makes them feel in a positive way. For example, participant SW responded that

.... *a gratitude book is with me, a paper or a diary I keep every time with me. Whatever good happens to me, I write, thank god for this, thank god for this, thank god for this, whatever small thing it was..... So, that makes me positive…when I am in distress or depressed, I read that and I get happy…. (PARTICIPANT 1, SW)*

Participants also perceive cancer as an opportunity to reflect and consider it as a signal to begin caring for oneself. Through this experience, patients try to see life from a different perspective and enjoy each moment of their life to its fullest. For example, participant SD reported,

*To be frank, this thing helped me change my perspective a lot. I would say that my attitude has changed after it happened to me.. it changed me as a person and made me a better person.. (PARTICIPANT 4, SD)*

### Theme 5: Individual Factors

One of the important factors that have been observed to influence is the individual's internal self. While taking care of oneself emotionally, both external and internal factors are influenced. Based on the internal makeup of the individual, they perceive a situation, act, and deal accordingly. Four categories were identified, which include (1) self-efficacy, (2) questioning and blaming self, (3) modeling, and (4) perceiving oneself as normal individual.

#### Self-Efficacy

Self-efficacy, or believing that one can overcome the disease, is an important factor that will help them endure harsh treatment which also increases their chances of survival. For example,

*Without belief in yourself or without will power, we can't work. So, this is the way we are dealing with our emotions or any type of problem or situation, our own beliefs are very important for all the problems…. (PARTICIPANT 6, BK)*

Participants reported that having a strong belief in oneself creates confidence in themselves, which helps them to be emotionally strong. The patients understand that this is one phase of life, which adds to their acceptance of the situation and the disease.

*.... Emotional self-care practices happen when you believe in yourself, you need to have confidence that everything is going to be alright.... Belief in self is very important…. (PARTICIPANT 9, KM)*

#### Questioning and Blaming Self

Participants reported that after receiving the diagnosis report, they begin to question why everything is happening to them, develop a negative attitude toward their life, and begin blaming themselves for being sick. For example, participant DS responded,

*I had phases and times where I really used to question myself how long it will take to get my hair back, whether life will still be the same, but then I pushed myself and made sure that I will not be in the loop of those negative thoughts…. (PARTICIPANT 10, DS)*

#### Modeling

Modeling is how a patient learns and is motivated to engage in proper health care by observing others. When they meet individuals who are improving both mentally and physically through active engagement in self-care behaviors, the participants report that they will begin to follow suit, which will have a positive impact on the patient's emotional self-care behaviors.

*If I see someone doing good, doing proper activities for his or her own self-care and indulging in some or other activities and it brings a positive effect on his or her life then, it would influence me to do that, and I would definitely follow it as well…. (PARTICIPANT 1, SW)*

Participants also reported that modeling also gives a patient another way to understand perceive the condition.

…*when we try to see a person from a different perspective, we look at the world according to our perspective… So when we step out we look at it in a different way…. (PARTICIPANT 10, DS)*

#### Perceiving Oneself as a Normal Individual

Some patients reported that even after understanding and knowing the diagnosis, the patient perceives themselves as a normal person and does not give much thought to being ill which helps them to stay more positive and also helps them to identify themselves as being a part of society.

*If something like that happens you have to stop thinking about it and keep yourself completely engaged. Should not be like sitting in a corner saying that I am having this disease and I am not able to do anything and my life is at an end, I don't have anything to do. You should avoid such kinds of thoughts..…. (PARTICIPANT 8, TN)*

### Theme 6: Socioeconomic Factors

As a social being, several factors, such as level of education, residence, and financial income, play a role in appropriately managing the condition. According to participants, these factors can also affect their outlook on how well they can emotionally manage themselves and take good emotional care of themselves. This theme particularly identifies such influences that are made by the individual's external characteristics. Three major sub-themes are identified which are (1) financial stability, (2) education, and (3) occupation.

#### Financial Stability

Participants have identified that having financial stability is an essential part when it comes to the treatment part of cancer. The available treatment options are prohibitively expensive. Once the individual feels secured in terms of the financial aspect, participants reported that they need not keep worrying or preoccupied with the future treatment and hence will be able to follow emotional self-care, for example,

*A financially wellsettled person might have better emotional self-care and there are too many people to support, so that person will be in better emotional self-care, so that will also have an impact..…. (PARTICIPANT 2, RH)*

#### Education

According to the participants, education provides wisdom and knowledge of the world from a different and more mature perspective. With good education, individuals become well aware of the various treatments available. Some participants identified that they were able to take care of themselves as a result of education, and this has an impact on their emotional self-care practices.

*I feel that through my education I was probably able to understand myself… probably understand my emotion and probably how to control my emotions and how to deal with it......... (PARTICIPANT 11, DS)*

Education gives an individual a power of confidence and self-acceptance, which will help them in accepting their condition and also helps in dealing with them.

#### Occupation

The occupation and its specific nature also influenced emotional self-care practices. The respect that the society gives an individual with a job will help them to gain self-confidence. Further, the financial stability that the job provides was reported to have influenced their self-care practices.

*Because I have a job we need not have a setback for aversion to going inside and speaking to someone. This is all an experience for us. My job was actually very positive for me. It has given me a lot of confidence to look......... (PARTICIPANT 8, TN)*

### Theme 7: Cultural Factor

Through this study, another factor that was found to influence emotional self-care practices was cultural influences. There are a lot of indigenous cultural practices that a person is supposed to follow, which correspond to different sets of laws and regulations of the religion they follow or the ethnic group they are part of. Participants have identified that cultural factors make a difference in their levels of engagement in emotional self-care practices. Two subthemes were identified which are (1) cultural norms and (2) cultural practices.

#### Cultural Norms

The specific set of rules and regulations of the ethic group or community that a person adheres to are cultural norms. This ranges from normal appearances to various dietary habits and lifestyle choices. These norms are sometimes aimed at patients as well, which has a negative impact on their emotional self-care practices. When an individual identifies themselves within a specific community, they follow and abide by its norms, not though but to an extent.

*Being rigid with rules and regulations affect you in a negative way because when you are emotionally weak, people around you and your family need to be flexible with what they follow, like they should think if you are able to follow it at that time or not, but being rigid in those rules and regulation is the negative part during those hard times…. (PARTICIPANT 11, DY)*

#### Cultural Practices

Unserviceable cultural practices by our forefathers have very less priority and importance in the current scenario. Following such cultures even now would bring in more anger and negative feelings, and hence effective management of emotional self-care becomes difficult with different physical conditions. This kind of practice also stigmatizes women, and more restrictions are imposed on them. This also brings in negative influences, such as guilt and shame. For example, participant SW pointed out,

*In a Brahmin family, if you are on your periods and even if it is in the late midnight also, you need to take a proper bath and need to shampoo your hair, which I don't like ok… the reason is that I am not healthy and my body is weak so how can I just force myself for a tradition or a bad practice of my forefathers to wash my hair in the middle of the night in winters and get ill and have a number of medicines which is going to make my body weak. I don't follow that custom…. (PARTICIPANT 1, SW)*

### Theme 8: Spiritual Factors

Spirituality is one of the factors seen to have an influence on the individual's emotional self-care practices. While understanding the influence of spirituality, even though the participants come from a specific religious background, they responded that following a specific religion and beliefs does not create any difference in emotional self-care. Rather, it is an individual's belief in the God entity and being one with God which led them to seek more positivity and improve their emotional self-care practices in a positive way. Two subthemes were identified under this theme, which are (1) mental relaxation and (2) spiritual beliefs.

#### Spiritual Belief

Along with the mental peace and calmness that they have, participants reported the belief that they have in their superior, which helps them to have a sense of hope and support and feel that God will be protecting them in such a vulnerable time. This belief instills hope and confidence in them, which helps them to see the disease condition more positively which in the long run, will help them in engaging in emotional self-care.

*it is my conviction that he (God) will help me... (PARTICIPANT 7, SIL)*

#### Mental Relaxation

Participants reported feeling calm and relaxed after engaging in meditative practices, such as chanting mantras or being in the presence of the supreme. This relaxation provides them with mental peace, which aids them in having better control over their emotions. For example, participant AM reported that,

…*.. We have a lot of Mantras and those are very powerful mantras. When you chant those mantras, you will definitely get positivity …. (PARTICIPANT 15 AM)*

## Discussion

Cancer treatment necessitates both an understanding of its symptomatology and the provision of specific medical treatments for better management. In addition to the medical treatment procedures, research evidence has also found several psychosocial interventions that are effective to face the challenges that a patient encounters during the treatment process and afterward (Park et al., 2019). The primary goal of this study was to better understand the various factors that influence cancer patients' emotional self-care behavior. Wang et al. (2019) explained that though the individual desires to have an optimistic view of their life several factors promote and hamper them from engaging in this behavior. The factor identified from this study which adds on to the existing knowledge is discussed here.

### Physical Factors

According to participants, treatment side effects could have a significant impact on their ability to adhere to emotional self-care routines. The intense pain and treatment's impact on the patient's body result in permanent changes and traumatic memory. Experiencing such profound effects makes it difficult for them to change their emotional self-care habit, and the majority of patients believe that this will have a negative impact on their emotional health. Niedzwiedz et al. (2019) pointed out that there can be a potential impact on patients' mental health when there are long-term mitigating effects owing to treatment load and uncertainty in numerous strata of their lives.

It has also been found that age and the maturity of individuals also count in their emotional experiences. In accordance with this study findings, patients who are aged found it easier and more prudent enough to take care of their own emotional health. Evidence indicates that age stands the moderating factor in terms of dealing effectively with their psychological distress (Zhang et al., 2020). The majority of participants reported that as they grew older and gained more experience, they gained a better understanding of the situation and were able to care for themselves emotionally and physically.

Gender was another factor found in the study that influenced the extent of which the individual engages in emotional self-care behaviors. In the study, most participants reported that, compared to men, women were better able to deal with their emotions and take care of their emotional health. Research indicates that there are significant gender differences that exist in understanding several conditions, such as depression and anxiety among patients with cancer (Pham et al., 2019). In terms of the pain tolerance and management of their conditions, it was seen that the female population were more tolerant compared to male population (Hinz et al., 2019).

### Social Factors

Society plays the vital role in improving and rehabilitating patients with cancer during and after treatment. There is a collectivist cultural trend in India that diminishes the role of society in an individual's life (Oyibo, 2021). The attitude of society toward such disorders and how they deal with such situations have a significant impact. According to the participants, the social avoidance and neglect that individuals face from society had a negative impact on their emotional self-care behavior. Because of a lack of understanding and awareness, society frequently views cancer as a communicable disease and avoids patients in social situations. This is a societal attitude that has a negative impact on the patient's emotional wellbeing and frequently leads to disorders, such as depression and anxiety (Pan et al., 2019). In pointing out the lack of awareness and information about cancer and relating this concept to Indian culture, the community predicts a large interconnection, which they link to Karma. In Indian tradition, terrible life experiences and meta-beliefs are generally attributed to the belief in Karma. Mishappenings are linked to God's will and fate in Indian literature (Dalal, 2020).

### Family Factors

The individual's immediate society is his or her family. All of the significant units in this group have a significant impact on the individual in terms of enhancing and engaging in emotional self-care behaviors. Participants in the study reported that the support and companionship they receive from their family is very important in terms of their emotional wellbeing. When an individual is placed in a non-suppurative setting, coping with the situation becomes challenging, compromising the individual's emotional wellbeing (Curigliano et al., 2020). According to the researchers, family members' perceptions of support have a critical role in coping with various negative emotions during the treatment and diagnosis process (Nuraini et al., 2018).

Another significant factor identified in the study was the spouse's support. Their corresponding spouse's companionship and support were found to significantly impact their emotional self-care routines. To overcome the challenges that they faced, spouses were considered as a supportive and inspiring unit within themselves. Evidence demonstrates that emotional support provided by their spouse aids the individual in overcoming a variety of emotional and psychological issues (Lee et al., 2018).

Individuals' interactions with emotional self-care were influenced by their upbringing and the environment in which they were raised since childhood. The environment in which they were frequently exposed aided them in adequately dealing with a variety of problems, and it was thought that improving their emotional health would be much easier. Parenting practices and the environment in which they grew up contribute to shape their attitudes and impressions of a variety of conditions that are viewed as bad. Having the mindset to overcome adversity is developed in an individual due to such experience and practice (Anthony et al., 2019).

### Psychological Factors

The individual's psychological makeup was also observed to have an impact on their engagement in emotional self-care actions. The psychological characteristics indicated by the participants had an impact on their emotional self-care participation, both positively and negatively. This study discovered the negative impact that many emotional apprehensions can have on people's participation in self-care routines. Cancer treatment is frequently ambiguous in terms of its impact and course after therapy. Even if the treatment appears to be effective, the individual is often plagued by a fear of recurrence.

When people live in constant fear, it affects their emotional state, which can lead to intrusive thoughts that prevent them from engaging in emotional self-care routines (Cupit-Link et al., 2018). Previous research shows that the diagnosis of cancer and the treatment process also have a comorbidity with certain conditions, such as depression and anxieties. There are many external and internal factors that can cause negative emotions, and having too many of them can have a negative impact on an individual's emotional self-care practices (Kennifer et al., 2009).

Stress is one of the most common and documented factors that influence emotional self-care behaviors and these unpleasant feelings. Individuals' psychological stress has been linked to the tumor's initiation and progression (Soung and Kim, 2015). Psychological stress is a determinant that has a significant impact on their viewpoint and interactions with one another during treatment (Levkovich et al., 2018).

Psychological factors are also acknowledged as important in eliciting pleasant feelings to optimize emotional self-care practices. The positive emotions identified by the individual assisted them in maintaining an optimistic outlook and taking a positive step during the course of cancer. According to researchers, individual's optimism and emotional stability are related to each other, if people have optimism, his or her quality of life would improve (Finck et al., 2018). Optimism has been reported to be a strong predator of depression in various studies, and it has also been demonstrated to be a helpful coping technique among patients with cancer of all countries and ethnicities (Fasano et al., 2020). Gratitude was identified as a component that aids the individual in bringing a positive attitude to their situation, and they were able to improve and engage in emotional self-care behaviors more optimistically with the virtue of gratitude (Sztachańska et al., 2019).

### Individual Factors

From the identified themes, one of the factors that influenced emotional self-care practices is individual factors. Individual self-efficacy is essential when it comes to engaging in emotional self-care activities. The presence of a strong conviction and belief in one's own ability to engage in activities promotes physical and emotional wellbeing, and it acts as a triggering element in determining the amount of time and quality of emotional self-care practices that an individual engages in Chin et al. (2021). When a person is given a diagnosis, they go through a phase of struggle before accepting it. However, the individual's mind will always be preoccupied with blaming actions and questioning the fate. This has a negative and destructive impact on self-efficacy, leading to degenerative practices in terms of emotional self-care on the inside (Doble et al., 2020). The study discovered that questioning and blaming oneself was one of the most common answers presented to the participants to clarify that most of the participants had gone through and had a negative impact on their emotional self-care behavior. Emotional self-care is developed not only by the individual but also by the influence that others have on them. Modeling someone who actively engages in emotional self-care activities encourages others to engage in such health-promoting behaviors on their own, allowing them to care for themselves both physically and emotionally (Lin et al., 2019). The difference between the individual's condition before and after treatment is enormous. To assist the treatment, people must make a variety of changes to their daily routine (McGeechan et al., 2022). Even when there is a significant difference between what was and what is now, participants observed that people who consider themselves to be patients frequently lose strength and capacity and are perceived as weak. Individuals who see themselves as normal people who go about their daily lives as they always have instilled greater power and confidence in themselves to overcome their circumstances (Kohi et al., 2019).

### Socioeconomic Factors

Socioeconomic factors were elicited to understand its influence on the engagement of emotional self-care practices. According to the findings, when insurance and other agencies take care of financial platforms, there is less tension and concern than when people lack financial assistance and stability. This intriguing aspect links specific predisposing ideas to poor emotional health, resulting in a reduction in the quality of emotional self-care actions that the individual engages in Carrera et al. (2018). Another factor that was identified to influence the emotional self-care behavior was education. Individuals' awareness of the importance of self-care and dealing with medical conditions, particularly understanding their conditions and how effectively they can deal with them, has significantly impacted their emotional self-care practices. Individual self-care was observed to improve and be applied more frequently when there was a presence of education and a clear idea for the individual self-care in general (Kohi et al., 2019). Connecting to an individual's financial stability and occupation is also important in emotional self-care techniques. Being in a work environment allows individuals to improve their personal and professional lives by demonstrating genuine concern and adopting a normal-life attitude. Occupation improves a person's financial situation and makes them feel strong and supported by their coworkers and their work (Murfin et al., 2020). When a person has a positive view, the amount of time working on their emotional health can increase.

### Cultural Factors

As the study is conducted in India, the cultural diversity among the specific geographical areas has an influence on certain behaviors. The wide variety of cultural aspects, religious beliefs, and norms that the society follows differ accordingly. Stemming from different mythological and religious texts, there are specific norms and thoughts which are beneficial as well as detrimental in nature (Bose, 2012). Participants in the study stated that following the cultural norms and practices is difficult and infuriating when there is societal pressure to conform to certain norms and practices. These cultural norms and practices often go too far, affecting an individual's emotional self-care. Several norms and practices will not agree or go along with the idea of emotional self-care when there is a complete shift in how an individual seeks time away from society and takes care of themselves emotionally (Williams et al., 2016). This creates a negative outlook and hinders the individual's ability to engage in a variety of emotional self-care practices that actually would help them to deal with their conditions and pain.

### Spiritual Factors

In terms of emotional self-care, an individual's belief in a higher power has been found to be an influencing factor and a supportive structure. The spiritual conviction gave them hope that they would be able to overcome their serious medical problem. Belief in spiritual powers, such as God, instilled a sense of optimism in patients. Prior evidence showed that an individual's faith in God had a major influence on shaping behaviors, leading them to engage in a variety of activities that assisted in improving their living situations through the developed and spiritual health locus of control (Meadows et al., 2020). Individuals' meditative practices and characteristics were also found to provide them with a sense of calmness and relaxation, allowing them to improve and engage more effectively in their emotional self-care, resulting in quality emotional wellbeing (Nuraini et al., 2018).

### Limitations of the Study

One major limitation of this study is the sample chosen focusing on patients with cancer in general, with participants majorly diagnosed with breast and lung cancer. Hence, more unique information could have been gathered by focusing on specific cancer types. The study was conducted amidst the COVID-19 pandemic, therefore, the researcher had chosen the telephonic interview method, and the major limitation of this method was that it restricts the interviewer from observing nonverbal responses. As this study used a purposive sampling method, it restricts the findings from generalization. Hence, future studies can focus on understanding cancer-type-specific self-care behaviors to accumulate precise information. The lack of participation encountered during the initial data collection process is also one of the limitations of the study. Due to this, the researchers could not collect data from the large sample size.

### Conclusion and Future Directions

Emotional self-care is a significant aspect that studies have identified as an important part of proper symptom management. This study identified the various factors that influence emotional self-care among Indians. In a pandemic-stricken era, being more at risk for infection and lack of proper accessibility to health care, a patient with cancer was not able to get proper health attention (Wang et al., 2021). Currently, illness management is encouraged to be done by the self; this knowledge assists health workers in developing effective cancer management techniques by improving the component of emotional self-care. When the patient engages in self-care practices, they should also be physically and emotionally at equilibrium to have symptom management. But most of the available interventions and studies have given an extreme focus to physical self-care, leaving out the emotional self-care aspects. In the future, incorporating this study findings' specific measures to understand emotional self-care behaviors and engagement can be formulated. Also, new effective interventions can be formulated by involving emotional self-care, so that effective self-management can be catered. This study findings also focus on how the caregiver and the society should be trained and made aware of these influencing factors to adopt holistic management by engaging the patient and the society to bring about a difference in the patients' lives. Incorporating this information, professionals working in this field can formulate productive plans and techniques to enhance holistic self-care practices. Services can be rendered through several self-help awareness classes for the patients along forming self-help groups among patients to enhance and incorporate self-care techniques into their treatment regime. This technique will also provide psychoeducation to society on how to respond to the treatment and diagnosis of cancer.

## Data Availability Statement

The raw data supporting the conclusions of this article will be made available by the authors, without undue reservation.

## Ethics Statement

The studies involving human participants were reviewed and approved by Ethical Review Board for the Protection of Human Subjects in Research, Central University of Karnataka, Kalaburagi, India. The Ethics Committee waived the requirement of written informed consent for participation.

## Author Contributions

AS has contributed to the conceptualization of the study, investigation, validation of data, and writing of the manuscript. ER has contributed to conceptualization, methods, supervision, and reviewing. RJ has contributed to conceptualization, methods, validation of data, and reviewing. MD has contributed to data analysis, reviewing, and writing of the manuscript. AG contributed in supervision and reviewing. RG has contributed to the data analysis. TJ has contributed to the preparation and editing of the manuscript. All authors contributed to the article and approved the submitted version.

## Conflict of Interest

The authors declare that the research was conducted in the absence of any commercial or financial relationships that could be construed as a potential conflict of interest.

## Publisher's Note

All claims expressed in this article are solely those of the authors and do not necessarily represent those of their affiliated organizations, or those of the publisher, the editors and the reviewers. Any product that may be evaluated in this article, or claim that may be made by its manufacturer, is not guaranteed or endorsed by the publisher.

## Appendix

### Interview Schedule

#### Self-Care Awareness

1. What is your idea/viewpoint about self-care behaviors?
2. According to you can you explain what are self-care practices and self-care behavior?
3. What do you think emotional self-care can be? Can you elaborate on it?
4. Can you explain about what could be the possible difference between physical and emotional self-care according to you?

### Factors Influencing Emotional Self-Care

#### Psychological

5. How do you think that your emotional states and your emotional wellbeing influence your emotional self-care practices?
6. Can you explain about your opinion about the influences that are made in your self-care practices by several negative feelings, such as fear, anxiety, depression, and feeling of uncertainty?
7. According to you do, can you explain whether that the belief that you have in your abilities actually influences your emotional self-care practices?
8. How do you think that self-image and self-identity can influence your emotional self-care practices?
9. Do you think the level of stress that you encounter actually can bring about a influence in your emotional self-care practices?

#### Social and Cultural

10. Can you elaborate on your viewpoints about the influence that your society play on emotional self-care practices? Do you think the amount of social support that an individual receive plays a role in their emotional self-care practices?
11. Around you, if there is a presence of someone who engages in a proper self-care regime actually influence you in engaging in self-care practices and why?
12. Do the culture and the ethnic group you belong to, its rules and regulation, and its ethical standards actually influence your emotional self-care practices, can you elaborate why?
13. Does, the religion or the belief system that you follow have an impact on the emotional self-care practices that you follow and how do you think it happens?

#### Sociodemographic Variables

14. Do the age and the gender that you belong to influence your emotional self-care practices and can you explain why?
15. Can you elaborate on to what extend do think that your education and occupation play an important role in the emotional self-care practices?
16. What can be the influences of your socioeconomic states have an influence on your emotional self-care practices and can you explain why?
17. How do you think that your marital status determines your level of engagement in emotional self-care practices?
18. According to you, how do you think the type of residence that you belong to have an influence on your emotional self-care practices?
